# Evaluation of an optimized metal artifact reduction algorithm for flat-detector angiography compared to DSA imaging in follow-up after neurovascular procedures

**DOI:** 10.1186/s12880-019-0352-2

**Published:** 2019-08-14

**Authors:** Nadine Amelung, Volker Maus, Daniel Behme, Ismini E. Papageorgiou, Johanna Rosemarie Leyhe, Michael Knauth, Marios Nikos Psychogios

**Affiliations:** 1 Institute for Diagnostic and Interventional Neuroradiology, University Medicine Göttingen, Robert Koch Straße 40, 37075 Göttingen, Germany; 2 Institute for Diagnostic and Interventional Radiology, University Hospital of Jena, Am Klinikum 1, 07747 Jena, Germany; 3 Institute for Radiology, Südharz Klinikum Nordhausen, Dr. Robert Koch Straße 39, 99734 Nordhausen, Germany

**Keywords:** Angiography, Brain/ brain stem, Flat-detector CT angiography, Coiling, CT metal artifacts reduction

## Abstract

**Background:**

Flat detector CT – angiography (FDCTA) has become a valuable imaging tool in post- and peri-interventional imaging after neurovascular procedures. Metal artifacts produced by radiopaque implants like clips or coils still impair image quality.

**Methods:**

FDCTA was performed in periprocedural or follow-up imaging of 21 patients, who had received neurovascular treatment. Raw data was sent to a dedicated workstation and subsequently a metal artifact reduction algorithm (MARA) was applied. Two neuroradiologists examined the images.

**Results:**

Application of MARA improved image appearance and led to a significant reduction of metal artifacts. After application of MARA only 8 datasets (34% of the images) were rated as having many or extensive artifacts, before MARA 15 (65%) of the images had extensive or many artifacts. Twenty percent more cases of reperfusion were diagnosed after application of MARA, congruent to the results of digital subtraction angiography (DSA) imaging. Also 3 (13% of datasets) images, which could not be evaluated before application of MARA, could be analyzed after metal artifact reduction and reperfusion could be excluded.

**Conclusion:**

Application of MARA improved image evaluation, reduced the extent of metal artifacts, and more cases of reperfusion could be detected or excluded, congruent to DSA imaging.

## Background

Intravenous Flat-detector computer tomography (FDCT) has become a valuable tool in peri- and postinterventional image acquisition after neurovascular procedures [1, 2]. It has been shown, that FDCT is a reliable method in follow-up examinations after neurosurgical clipping of aneurysms and aneurysm coiling with or without stent placement [3]. Metal induced artifacts like streak or hardening artifacts impaired evaluation of aneurysm reperfusion, adjacent brain tissue and parent vessels in post-interventional images. Recent improvements in metal artifact reduction algorithms (MARA) led to a significant improvement in image appearance and post-interventional evaluation [2–5]. Previous studies have shown that application of a dedicated MARA in post-interventional FDCTA improves evaluation of aneurysm reperfusion, parent vessels and small vessels in the level of the implant as well as adjacent brain tissue [6].

Current studies have been performed comparing original and MARA optimized datasets of FDCTA [4, 7]. This study is going to compare original and MARA optimized data against the current gold-standard digital subtraction angiography (DSA).

## Methods

### Patients

Over a period of 3 months, 21 patients have been examined via flat detector computer tomography angiography (FDCTA). Twenty-one patients had follow-up examinations via DSA as well as FDCTA with and without MARA. Of those, 13 patients received follow-up examinations after endovascular coiling, 2 patients had intracranial clips, 3 patients had intracranial clips and coils, 1 patient underwent intracranial clipping and stent assisted coiling, 1 patient had a flow diverter and a clip, and 1 patient received FDCTA after stent-assisted coiling.

According to the guidelines of the local ethics committees, ethic approval was given for the retrospective evaluation of patient data, which was conducted in accordance to the Declaration of Helsinki.

### Image acquisition

Images were acquired on an angiography suite (Artis Q Angiography System; Siemens Healthcare GmbH, Forchheim, Germany) immediately after patients underwent DSA for follow-up after endovascular or neurosurgical therapy. If DSA and rotational angiography were performed simultaneously, the 5-s protocol was performed. If FDCTA was performed as a separate examination and DSA was performed later, we applied a 10 s or 20 s protocol. The 10-s protocol was used only for clips or coils, the 20-s protocol was applied to patients after stent-assisted coiling.

Each protocol covered a rotational angle of 220°. The 5-s rotation protocol, resulting in 133 projections was performed with arterial injection of contrast agent. The 10 or 20 s protocol resulted in 248 and 496 projections, respectively, and was performed with intravenous injection of contrast agent. After injection of contrast agent the rotation was started manually after bolus tracking in the carotid siphon. Sixteen patients received a 5 s rotation protocol with intra-arterial injection of 16 ml of contrast agent (Imeron 400, Bracco Altana Pharma, Konstanz, Germany) at a flow rate of 2.4 ml/s via a 5F - diagnostic catheter in the anterior circulation. Two patients were treated in the posterior circulation. They received an intra-arterial injection of 12 ml contrast agent at a flow rate of 1.8 ml/s.

Five patients received a 10s rotation protocol and 2 patients received a 20s rotation protocol. Those protocols included application of 40 ml contrast agent (Imeron 400, Bracco Altana Pharma, Konstanz, Germany) followed by 40 ml of saline flush injected into a cubital vein at a flow rate of 5 ml/s. Fluoroscopy imaging was used for bolus tracking before starting the 10s and 20s protocol. The 3D dataset was processed on a designated Siemens Workstation (Syngo X Workplace, Siemens, Forchheim, Germany). For reconstruction a “sharp” reconstruction kernel was applied, images had a 512 × 512 pixel matrix and an isotropic voxel size of about 0,3 × 0,3 × 0,3 mm.

### Metal artifact reduction

The metal artifact reduction algorithm is a prototype based on the normalized metal artifact reduction algorithm, which has been described before [1–3, 6]. Except for minimal changes in the optimization process, the algorithm remained unchanged, compared to earlier studies [1–3, 6].

Raw data was processed on an offline research workstation by Siemens. The metal artifact reduction algorithm consists of several steps. At first, an uncorrected volume is calculated. In this volume the metal containing implants, either clips or coils, are identified and the correction process is started. After segmentation a binary metal volume is created.

Forward projection of the binary metal volume yields a projection image of metal regions on the detector in each position. These metal bearing regions are responsible for the artifacts, thus the data itself and on the boundary line must be replaced. Then normalized projection images are computed by relating (dividing projection images by uncorrected volume) projected and uncorrected images to each other. The masked metal in the normalized projections is removed by interpolation and the corrected projections are denormalized and reconstructed as described before [2]. Finally, data along the boundary line is smoothened and a procedure minimizing total variation is applied in order to reduce streak artifacts [2].

Multiplanar reconstructions (MPR) in standard projections and maximum intensity projections (MIP) were created using the datasets of original and MARA optimized data. Additionally to reconstructions using the standard field of view (FOV) of the whole head, a smaller FOV (256 × 256 pixel) was created containing the implant and the parent vessel as well as adjacent brain parenchyma.

### Image analysis

Corrected and original images were randomized. Two raters (D.B. and N.A.) evaluated the images on a designated Syngo X Workplace (Siemens, Forchheim, Germany). The raters could adjust window width and center manually. They could also rotate final images, use a smaller FOV and MIP images as preferred. The raters identified the implants and the target vessels. They evaluated the images concerning the extent of metal artifacts in general on a Likert scale from 1 to 4 (1 = no artifacts, 2 = a few artifacts, 3 = many artifacts, and 4 = extensive artifacts). They had to evaluate, whether the implant, residual perfusion of the aneurysm, restenosis in the parent vessel, stenosis in the area of the stent markers, adjacent brain parenchyma, adjacent small vessels, bleeding, and infarction could be evaluated on a scale from 1 to 3 (1 = yes, 2 = no, 3 = no evaluation possible). Adjacent brain parenchyma was defined as brain parenchyma within a radius of 2 cm around the metal implant. They had to evaluate how well the parent vessel, the ipsilateral adjacent brain parenchyma, the contralateral brain parenchyma as well as stent struts could be identified, on a scale from 1 to 4 (1 = excellent, 2 = good, 3 = limited, 4 = not possible). Ipsilateral brain parenchyma was defined as parenchyma of the same hemisphere as the metal implant. Contralateral brain parenchyma was defined as brain parenchyma of the contralateral hemisphere.

The corresponding DSA images were evaluated by the same criteria except for those cases, where this was impossible (i.e. infarction, evaluation of brain parenchyma). Raters could choose to use a smaller FOV, which might add some information regarding smaller structures like stent struts.

To avoid recall bias, data was divided into two groups, evaluated separately after an interval of 4 weeks.

### Statistical analysis

Interrater agreement was calculated with Cohen’s Kappa. In cases of disagreement a third experienced rater (M.P.) evaluated the datasets again. Original and MARA-optimized data were compared to DSA data. Statistical analysis included McNemar’s testing and chi-squared test for testing the results without MARA against he results with MARA concerning reperfusion at the aneurysm base, and Wilcoxon signed ranking test of data concerning evaluation of adjacent structures, ipsi- and contralateral brain parenchyma, stent struts, parentvessels, and the extent of artifacts. Results of both raters regarding reperfusion of the aneurysm without and with MARA were visualized on an ROC curve performed with MedCalc Version 12,1, MedCalc Software, Ostend, Belgium.

## Results

Twenty-one patients were examined via FDCTA. One of these patients received a 5 s rotational angiography and a 10s FDCTA, one patient received a 10s FDCTA as well as two 5 s rotational angiographies, one patient received a 5 s rotational angiography and a 20s FDTCA, and one patient received a 10s and a 20s FDCTA thus the examinations add up to more than 21. In Table 1 indication and type of examination are listed for each patient.

**Table 1:** Indication and type of examination are listed for each patient

Patient	Target Vessels	Metal Implants	Protocol
1	ACom	clip, coiling	iv FDCTA 10s
2	ACom	coiling	5 s rotational angiography
3	ICA	flow diverter, clip	iv FDCTA 10s
		flow diverter, clip	iv FDCTA 20s
4	ICA	clip, stent-assisted coiling	iv FDCTA 10s
5	ACom	coiling	5 s rotational angiography
6	ACom	coiling	5 s rotational angiography
7	VA	coiling	5 s rotational angiography
8	ICA	coiling	5 s rotational angiography
9	MCA	clip	5 s rotational angiography
10	MCA	3 clips, coiling	iv FDCTA 10s
11	ACom	coiling	5 s rotational angiography
12	ACom	coiling	5 s rotational angiography
13	ICA	coiling	5 s rotational angiography
14	ACom	coiling	5 s rotational angiography
15	ACom	stent-assisted coiling	5 s rotational angiography
		stent assisted coiling	iv FDCTA 20s
16	ICA	2 clips, coiling	5 s rotational angiography
17	ACom	coiling	5 s rotational angiography
18	ACom	coiling	5 s rotational angiography
19	ACom	coiling	5 s rotational angiography
20	ACom	coiling	5 s rotational angiography
21	ICA and PCA	3 clips	5 s rotational angiography
		3 clips	iv FDCTA 10s
		3 clips	5 s rotational angiography

*Abbreviations*: *ACom* anterior communicating artery, *ICA* internal carotid artery, *VA* vertebral artery, *MCA* middle cerebral artery, *PCA* posterior cerebral artery

**Fig. 1:**
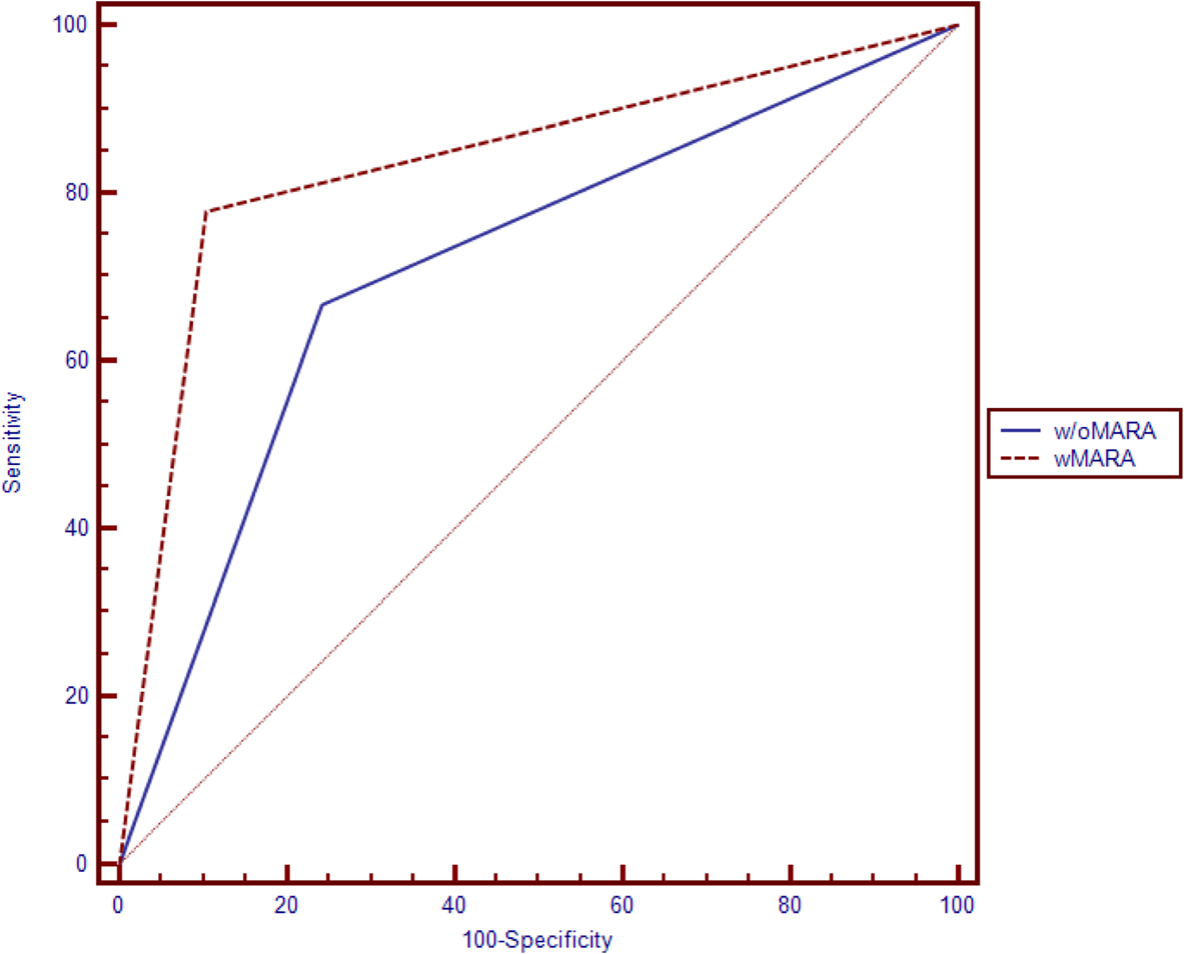
ROC curve of the results of both raters concerning detection or exclusion of reperfusion before and after application of MARA. Application of MARA improved sensitivity

**Fig. 2:**
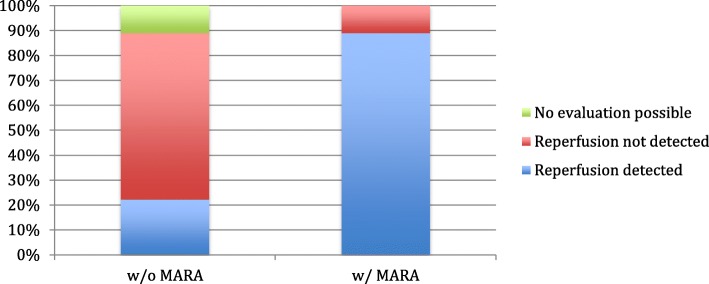
In those cases, where reperfusion was diagnosed via DSA, application of MARA increased the detection of reperfusion. Eight out of ten cases of reperfusion were diagnosed correctly after application of MARA, whereas only 6 cases were diagnosed in the images without MARA. Additionally there were 2 cases, in which reperfusion should have been detected but images without MARA were not diagnostic

Application of MARA significantly changed the detection or exclusion of reperfusion compared to data before application of MARA (chi-squared testing: 0.0369). Application of MARA improved sensitivity of detection or exclusion of reperfusion, AUC without MARA 0,713, 95%CI 0,534 – 0,847, AUC with MARA 0,837, 95%CI 0,682–0,937 (see Fig. 1). In all FDCTA images it was possible to identify the implants correctly. The parent vessel could be named correctly in all cases. Application of MARA improved evaluation of the implants concerning identification of reperfusion significantly as opposed to evaluation before application of MARA (8/9 cases with MARA, 2/9 cases without MARA; *p* < 0.05). Nine cases of reperfusion could be identified with DSA. After application of MARA to FDCTA imaging, 8 cases of reperfusion could be identified (88%), whereas without MARA only 2 cases (22%) could be identified in FDCTA imaging (Fig. 2).

Only 2 (8%) datasets could not be evaluated concerning reperfusion after application of MARA as opposed to 6 (24%) datasets before. Among those FDCT images not possible to evaluate, were 4 (16%) cases without MARA and 2 (8%) cases with MARA where reperfusion should have been excluded according to DSA imaging.

The extent of artifacts was rated “extensive” in 3 out of 25 (12%) cases and as “many” in 12 out of 25 cases (48%) before application of MARA. After application of MARA only one case was rated having extensive artifacts (4%), and 7 were rated having “many” artifacts (28%).

Application of MARA to FDCTA imaging reduced the cases where no statement concerning parent vessel stenosis, 8 (32%) with MARA instead of 9 (36%) without MARA, stenosis near stent borders, 1 (4%) with MARA instead of 2 (8%) without MARA, or adjacent small vessels, 1 (4%) with MARA instead of 2 (8%) without MARA could be made. Bleeding detection as well as identification of infarction or small vessels nearby was not changed significantly.

**Fig. 3:**
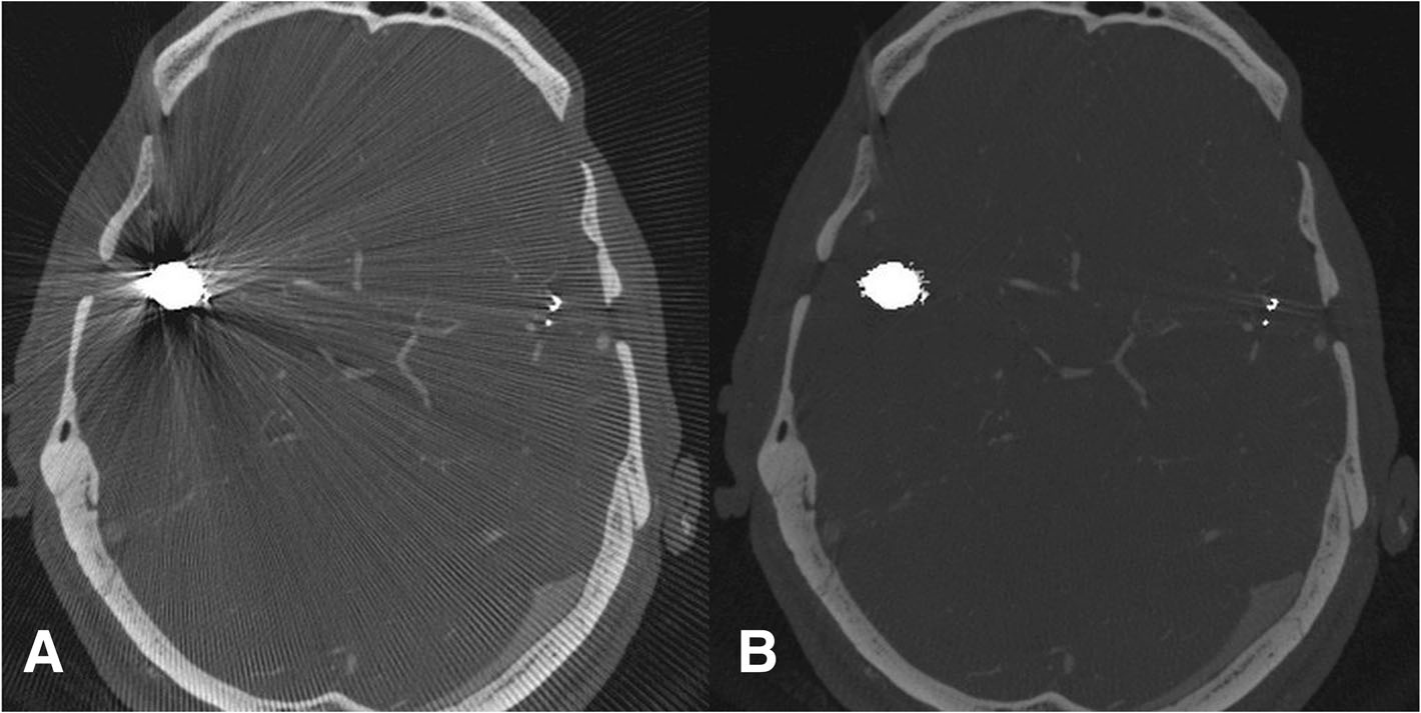
In image **a** multiple streak artifacts impair evaluation in the level of implant, whereas in image **b** after application of MARA the image quality is improved significantly and the streak artifacts are eliminated

In general, assessment of parent vessel, adjacent brain parenchyma, as well as ipsi- and contralateral brain parenchyma was rated superior in MARA-corrected images compared to original data (see Fig. 3).

Concerning the evaluation of parent vessels, 13 (52%) of the MARA optimized images were rated superior to the non-optimized images, resulting in a rating of “good” or “excellent” as compared to “limited” or “not possible” before MARA. In 11 cases (44%) application of MARA led to a superior rating of adjacent brain parenchyma. Eight cases (32%), which were rated as “limited” or “not possible” before application of MARA were rated as “good” after application of MARA. Two cases (8%) were rated as “good” before application of MARA, after application of MARA these cases were rated as “excellent”. In one case (4%) application of MARA improved evaluation of adjacent brain parenchyma from “not possible” to “limited”. Evaluation of ipsilateral brain parenchyma was improved in 9 cases (36%). Without MARA those cases were rated as limited, after application of MARA ipsilateral brain parenchyma was rated as “good” in those cases.

Wilcoxon signed-rank test showed significantly improved assessment of contralateral brain parenchyma in 14 cases (56%). Without application of MARA evaluation of contralateral brain parenchyma was rated as “limited” in 9 cases (36%), after application of MARA contralateral brain parenchyma was rated “good” on those cases (*p* < 0.05). In 5 cases (20%) assessment of contralateral brain parenchyma was not possible before application of MARA, after application of MARA, limited evaluation was possible.

The extent of artifacts was rated significantly less severe in 16 cases (64%) after application of MARA compared to original data (*p* ≤ 0.001). There was no statistically significant difference in assessment of parent vessel and brain parenchyma of the ipsilateral hemisphere.

Inter rater agreement was 100% calculated by Cohen’s Kappa, concerning identification of implants, parent vessels and reperfusion in DSA images.

## Discussion

In this study, FDCTA images were evaluated before and after application of an optimized metal artifact reduction algorithm. Images were also compared to DSA – the current gold standard in follow-up of endovascular or surgical treated aneurysms.

As shown before [1, 3, 4, 6, 7], application of the improved MARA improved evaluation of FDCTA images in all cases. More images could be evaluated after application of MARA. After application of MARA, image quality was rated superior, the artifact load was rated less severe, and evaluation of adjacent as well as contralateral brain parenchyma improved. Soft tissue resolution could not be improved after application of MARA. Detection of infarction or bleeding did not improve significantly after metal artifact reduction compared to raw images.

**Fig. 4:**
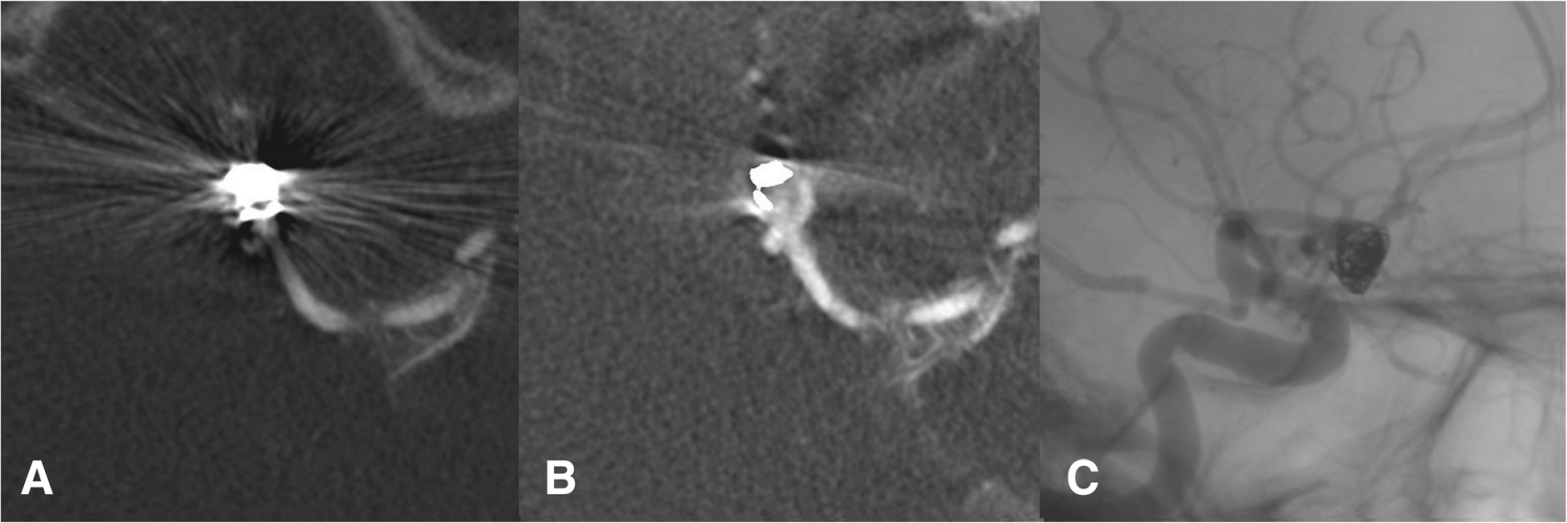
In image **a** a statement concerning residual perfusion of the aneurysm was not possible due to streak artifacts and image hardening caused by the coil material. After application of MARA the residual perfusion could be detected as seen in image **b**, congruent to the result of the digital subtraction angiography as seen in image **c**

It has been shown, that application of MARA improved evaluation of FDCTA images after endovascular or surgical intervention as opposed to unmodified data [6], the correlation with DSA images has not been made before. MARA improved evaluation of FDCTA images concerning reperfusion of aneurysms. A higher rate of reperfusion could be detected. In 6 cases, where reperfusion could not be diagnosed due to artifacts in FDCTA imaging without MARA, application of MARA showed reperfusion of the aneurysm, according to DSA images (see Fig. 4).

Without application of MARA, there were 4 cases (16%), where according to DSA imaging reperfusion should be excluded but due to extensive metal artifacts evaluation of FDCTA imaging concerning reperfusion was not possible. After application of MARA the raters were able to exclude reperfusion in 2 of those cases. Only 2 cases (8%) remained, where reperfusion could not be determined after application of MARA.

In other metal artifact reduction algorithms, it was reported, that application of MARA led to an alteration of data in the level of implant, primarily not affected by metal artifacts [2]. Due to these artifacts information regarding brain parenchyma and vessels in the level of implants is lost. Additional diagnostic might be necessary to gather this information. As shown before [4], the application of the improved MARA did not alter data not affected by metal artifacts. No information regarding brain parenchyma and vessels is lost after application of this improved MARA.

In 5 cases (20%), where patients had been treated with multiple clips or clips and coils, MARA could not be applied to all implants simultaneously. This led to persisting metal artifacts in the level of implants. A future improvement of a metal artifact algorithm might be the application to multiple implants simultaneously.

Additionally to the standard FOV of 512 × 512 pixel, a smaller FOV, containing only the implant and the adjacent parent vessel and brain parenchyma was calculated. This additional FOV was rated helpful in 10 out of 25 cases (40%) by both raters, regarding evaluation of stent struts or helping in the final decision, to exclude or diagnose reperfusion at aneurysm base.

This study was limited by its small sample size. Only 21 patients were included in this study. Depending on the implants used in each patient, the protocol differed. Three different protocols were used depending on the implants used in each patient and the time of the examination. Rotational angiographies were acquired during diagnostic catheter angiography, and intravenous FDCTA was acquired during follow-up examinations. A 5 s rotational angiography protocol with intraarterial application of contrast agent, a 10 s or a 20 s protocol with intravenous application of contrast agent were applied. Expectedly soft tissue resolution was best in the 20-s protocol and metal artifact load was least but also radiation exposure increased due to the increased number of projections. Application of MARA improved image evaluation in all applied imaging protocols. Further limitations were the variety of used metal implants and the different metals.

In cases of patient’s movement, high volume coils or multiple implants, the algorithm is still limited because residual artifacts may persist in the level of implants.

## Conclusion

Application of the optimized MARA improved image analysis after endovascular and surgical therapy of aneurysms. In cases, where metal artifacts made it impossible to evaluate reperfusion at aneurysm base, application of MARA improved images and quality of evaluation. In 6 cases, reperfusion of aneurysms could be depicted only after application of MARA, corresponding to DSA imaging. Extent of artifacts was reduced, whereas no significant information was lost i.e. concerning parent vessels or adjacent brain parenchyma as well as brain parenchyma in level of implant. FDCTA images added additional information, regarding implants located in other vessels, compared to DSA. Application of MARA leads to better evaluation of FDCTA images and can reduce the necessity to examine patients via DSA in follow-up examinations after neurovascular procedures.

## Data Availability

The datasets used and analysed during the current study are available from the corresponding author on reasonable request.

## Abbreviations

CI: Confidence interval
DSA: Digital subtraction angiography
FDCT: Flat-detector CT
FDCTA: Flat – detector CT angiography
FOV: Field of view
MARA: Metal artifact reduction algorithm
MIP: Maximum intensity projection
MPR: Multiplanar reconstruction
